# Long-Term Follow-up (14 to 25 Years) Following Closed Reduction and Early Movement for Simple Dislocation of the Elbow

**DOI:** 10.2106/JBJS.23.00288

**Published:** 2023-08-24

**Authors:** Thomas Mackinnon, Thomas D. Samuel, Edward Hayter, George Lee, Daniel Huntley, John Hardman, Raymond E. Anakwe

**Affiliations:** 1 Department of Trauma and Orthopaedic Surgery, St. Mary’s Hospital, Imperial College Healthcare NHS Trust, London, England; 2 Imperial College London, London, England

## Abstract

**Background:**

We have previously reported on the midterm outcomes after a nonoperative protocol to treat simple dislocations of the elbow that included a short period of splinting followed by early movement. We have now performed extended follow-up of the original patient group from the prior study to determine whether the excellent results that previously had been reported were maintained in the long term and also to determine the rate of and need for any late surgical intervention.

**Methods:**

We attempted to contact all of the patients from the original study group. We requested that they complete the Oxford Elbow Score (OES) survey, the Disabilities of the Arm, Shoulder and Hand (DASH) questionnaire, and a validated patient satisfaction questionnaire. Patients also were requested to attend a face-to-face assessment to have a clinical examination that included neurovascular, range-of-motion, and ligamentous stability assessments.

**Results:**

Seventy-one patients from the original patient group agreed to participate in the new study. The mean duration of follow-up was 19.3 years. At the time of the final follow-up, patients reported excellent functional outcome scores and a preserved functional range of movement in the injured elbow. The mean OES was 91.6 points, the mean DASH score was 5.22 points, and the mean satisfaction score was 90.9 points. None of the patients had undergone delayed or secondary surgery for instability during the interval period.

**Conclusions:**

This study demonstrated that the original excellent outcomes following treatment with a protocol of a short period of splinting and early movement remained excellent and were maintained into the very long term.

**Level of Evidence:**

Therapeutic Level IV. See Instructions for Authors for a complete description of levels of evidence.

The treatment for a simple dislocation of the elbow is generally accepted to be a short period of immobilization followed by early movement. Patient outcomes with this approach are generally reported to be excellent, although there is good evidence that the outcomes are not entirely benign. We performed a previous study that showed that a large number of patients reported some persistent pain following this nonoperative treatment protocol, although the pain was usually self-limiting and did not necessarily translate to further treatment^1^. A smaller number of these patients reported symptoms of persistent instability, and it has been shown that prolonged immobilization is associated with an increased risk of elbow contracture and stiffness.

Despite the general consensus regarding nonoperative treatment of most simple elbow dislocations, there has been continued debate about which injuries might benefit from early surgery and which simple dislocations of the elbow are best treated with primary ligamentous repair^2,3^. It has been suggested that subclinical instability may contribute to later pain, loss of function, and degenerative changes, and this may be a reason to advocate early surgery for patients with simple elbow dislocations, particularly those with high demands^4^.

We performed the present study to assess the extended outcomes for patients with a simple elbow dislocation who had been treated with a protocol that involved a short period of immobilization followed by early movement. We undertook this extended follow-up study to determine if the favorable early to midterm outcomes reported in the earlier study, and more generally in the literature, were maintained over time and into the very long term. We hypothesized that patient-reported outcome measures (PROMs), range of movement, and satisfaction would be preserved and would not show time-related deterioration. Our secondary hypothesis was that the rates of and need for late or delayed surgical intervention would be low.

## Materials and Methods

This secondary study was based on a previously reported study that examined PROMs following a nonoperative treatment protocol for a simple dislocation of the elbow. All of the patients had been treated with closed joint reduction, a short period of splinting, and early movement. The detailed methodology has been described previously^5^. The study was approved by our institutional review board, and each patient provided informed consent to participate. We wrote to all of the patients in the original study group to request that they participate in the new study. We requested that each patient repeat the PROM surveys and questionnaires that they had originally completed in the prior study (namely, the Oxford Elbow Score [OES]^6^ and the Disabilities of the Arm, Shoulder and Hand [DASH] questionnaire^7^) and to return these by mail. We also requested that each patient complete our previously validated patient satisfaction questionnaire^8^. Patients were asked to rate their level of satisfaction with the result of the treatment of their elbow injury on an anchored visual analog scale measuring 100 mm; the response was converted to a score between 0 and 100. The patient was also asked if subjective pain, stiffness, or instability persisted; the answer to each of these questions was recorded in the form of a dichotomous “yes” or “no” response. Nonresponders were sent a follow-up request after 1 month. Patients who agreed to participate were invited to attend a face-to-face outpatient appointment with an independent and trained assessor who was blinded to the earlier reported functional outcome scores and any previously collected data for each patient.

At the outpatient appointment, each patient underwent a full clinical examination of both elbows. The range of movement was assessed using a standardized protocol with a stainless-steel long-arm goniometer. Flexion and extension were measured with the shoulder in 90° of forward flexion and the forearm in maximal supination. The long arms of the goniometer were aligned with the acromion and the radial styloid. Pronation and supination were measured with the shoulder in the neutral position and the elbow held in 90° of flexion. Each measurement was taken 3 times, with a minimum of 5 minutes between measurements, and the mean value was recorded. Measurements were rounded to the nearest 1°. The neurovascular status of the upper limb was evaluated, and the elbows were assessed specifically for instability with use of varus and valgus stress tests performed at 30° of flexion. The pivot shift test was performed to demonstrate any posterolateral instability.

The primary outcomes were the changes in the OES and the DASH score over time. Secondary outcomes were the change in the elbow range of movement and level of patient satisfaction over time, as well as any cases of delayed or secondary surgery to address symptoms of pain, stiffness, and/or instability.

### Statistical Analysis

Histograms were used to confirm the normality of the continuous variables. Paired t tests were used to compare the range of motion in the injured and uninjured elbows for each patient. In addition, the change in the range of motion of the injured elbow was compared over time. The OES and each of its components were scaled to range from 0 to 100 points. The DASH score, the OES, and the patient satisfaction score were expressed as the mean and 95% confidence interval (CI). A chi-square test was used to compare the dichotomous variables. Descriptive statistics are presented for all of the patients who participated in the study. Demographic data, injury and treatment patterns, and the original OES and DASH scores were studied to compare the patient group in the present study with nonresponders and patients who declined to participate in the study. Values were expressed as the mean and 95% CI unless otherwise specified. A p value of <0.05 was considered significant.

### Source of Funding

There was no external funding for this study.

## Results

Seventy-one patients (64.5%) from the original study group agreed to participate in the new study, which included completing outcome instruments and attending a face-to-face appointment. Of these, 40 patients (56.3%) were men. The mean patient age was 46.9 years (range, 26 to 82 years; standard deviation, 16.5 years). The mean age for men was significantly younger than that for women (43.4 compared with 52.5 years; p < 0.001). We confirmed that 15 patients (13.6%) from the original study group were deceased. There was no demonstrable difference in patient age, sex, injury mechanism, and the original PROMs between the patient study group and the patients who were nonresponders or declined to participate.

In the study group, 52 patients used a splint for ≤1 week following joint reduction; the remaining 19 patients used a splint for ≤2 weeks. Physical therapy was prescribed for 60 patients, but records of attendance and the treatments that were provided were not available. The exact duration of splinting was dependent on the timing of outpatient follow-up. Physical therapy was prescribed at the discretion of the treating surgeon.

The mean duration of follow-up was 19.3 years (range, 14 to 25 years). At the time of the final follow-up, patients reported excellent functional outcome scores and preserved functional range of movement in the injured elbow. The mean OES was 91.6 points (95% CI, 89.4 to 93.8 points), the mean DASH score was 5.22 points (95% CI, 3.1 to 6.8 points), and the mean satisfaction score was 90.9 points (95% CI, 88.8 to 93.0 points). Univariate analysis confirmed that the OES, the DASH score, and the satisfaction score were all worse for women (Table I), and this difference was significant for all of these metrics with the exception of patient satisfaction.

**TABLE I:** Patient Demographics and Patient-Reported Functional Scores*

	Men	Women	P Value
No. of patients	40	31	
Mean age *(yr)*	43.4	52.5	<0.001
Mean PROMs			
DASH	3.3	8.9	0.015
OES (scale, 0 to 100)			
Overall	94.8	88.6	0.034
Pain	96.4	91.3	0.042
Functional	95.8	90.2	0.023
Psychosocial component	92.2	84.3	0.003
Satisfaction (scale, 0 to 100)	91.3	89.6	0.451

* PROMs = patient-reported outcome measures, DASH = Disabilities of the Arm, Shoulder and Hand, and OES = Oxford Elbow Score.

Overall, at the final assessment, the injured elbows continued to show small but persistent range-of-motion deficits when compared with the uninjured elbow (Table II). However, all of the patients did maintain a functional range of movement in the injured elbow^9^ and did not show any significant deficits in range of motion (elbow extension, flexion, flexion-extension arc, pronation and supination) compared with the uninjured side or further deterioration between the index and final assessments (Table II).

**TABLE II:** Clinical Range of Motion for the Injured and Uninjured Sides and Index and Final Assessments*

Comparison	Extension *(deg)*	Flexion *(deg)*	Flexion-Extension Arc *(deg)*	Pronation *(deg)*	Supination *(deg)*
Side-to-side difference in final measurements					
Injured side	7.4 (4.2 to 9.1)	134 (130 to 136)	130 (125 to 136)	82 (80 to 84)	82 (76 to 85)
Uninjured side	1.5 (0 to 4.5)	138 (135 to 140)	136 (130 to 140)	84 (83 to 85)	82 (78 to 85)
P value	<0.001	0.05	0.01	0.857	0.614
Change in injured side between studies					
Index measurement	8.1 (6.6 to 9.7)	135 (134 to 137)	128 (125 to 130)	83 (81 to 84)	83 (82 to 85)
Final measurement	7.4 (4.2 to 9.1)	134 (130 to 136)	130 (125 to 136)	82 (80 to 84)	82 (76 to 85)
P value	0.642	1.0	0.466	0.944	0.722

* The values are given as the mean and 95% confidence interval. P values were determined with a paired t test.

The OES, the DASH score, and the patient satisfaction score were excellent at the final assessment and demonstrated improvement over the interval between the studies. There was no demonstrable correlation of absolute range of motion with the OES (r = 0.308, p = 0.45), the DASH score (r = 0.226, p = 0.38), or the patient satisfaction score (r = 0.420, p = 0.25) at the final assessment.

None of the patients reported any additional episodes of instability or dislocation, and none reported subjective elbow instability or demonstrated any clinical evidence of instability on stress-testing or the pivot shift test. Twelve patients (17%) reported persisting subjective stiffness of the elbow. None of the patients had undergone delayed or secondary surgery for instability during the interval period. One patient (1.4%) had subsequently undergone a total elbow replacement after receiving a diagnosis of osteoarthritis. Nineteen patients (27%) reported persisting residual elbow pain, but all of these patients reported that this was intermittent and self-limiting.

When functional outcome scores were compared over time, the improvement in the OES and the DASH score for the injured elbow in the period between the original study and the final assessment was clear but not statistically significant. Patient satisfaction also improved over the interval period.

In the original study, 54 patients reported that they had participated in sport before their injury. We assessed 42 (78%) of those patients in our secondary study, and 36 (86%) reported that they continued to participate in their sport. Five patients in the original study had sustained a closed peripheral nerve injury, which affected the ulnar nerve in 2 patients and the median nerve in 3 patients. Patients with ulnar nerve injuries continued to note sensory symptoms at a mean of 13 years after injury, but these had resolved by the final assessment at a mean of 23 years.

## Discussion

Our study demonstrated that the excellent patient-reported and functional outcomes after treatment of simple elbow dislocations with a protocol of closed reduction, a short period of splinting, and early movement were sustained in the very long term. Patients showed modest further improvements in the OES, the DASH score, and patient satisfaction over time and into the long term.

The previous study had shown that, although the results of this treatment protocol were overwhelmingly excellent, the PROM scores for women were poorer; our secondary study demonstrated that this difference was maintained into the long term. The reasons for this are not clear, and it does not seem to be related to the objective range of motion of the elbow, which did not show any significant sex-related difference; thus, the cause is likely more complex than a direct relationship between sex and outcome. The women in our cohort were older than the men, and our original study showed that there were obvious differences in the mechanism of injury; however, our previous work showed that the differences in outcome scores for men and women persisted even when these factors were corrected for.

It is noteworthy that the mean OES, DASH score, and patient satisfaction score all showed significant improvement between assessments (at a mean of 7 years in the original study and a mean of 19 years in this study). The original study showed a clear relationship between absolute range of movement and PROMs and satisfaction. This effect was not maintained in our extended study. The patients showed further improvement in their outcome scores and satisfaction, but there was no significant change in objective range of motion; additionally, absolute range of motion in any plane did not prove to be a predictor of the OES, the DASH score, or patient satisfaction. It is likely that this reflects the complexity of what is encompassed in PROMs. It is well recognized that patient satisfaction is influenced by much more than objective function. The psychosocial elements of injury, treatment, and recovery are increasingly understood but difficult to separate from general outcome measures^10,11^.

None of the patients required delayed or secondary surgery for instability, and only 1 required joint replacement surgery due to osteoarthritis. We succeeded in following only 64.5% of the original patient cohort, so it is possible that pain, stiffness, and even instability may be underreported in our study. We recognize that this is a potential limitation of the study. Similarly, we recognize that the potential for bias related to patient selection, treatment, and local expertise was inherent in both the original and long-term follow-up studies, which means that our findings may not be generalizable.

Some patients continued to show persisting deficits in range of movement of the injured elbow in the long term. These deficits were generally small but were consistent and measurable when the injured and contralateral elbows were assessed, even in the very long term. The resulting deficits in range of movement were generally small and, as such, the statistical analysis was vulnerable to rounding errors—a further limitation of our study. In addition, it is not possible to make specific comments on the impact of physical therapy, which was prescribed at the discretion of the treating surgeon.

Persisting but mild residual pain and stiffness were common in the patient study group, but this did not seem to impact function and functional outcome scores. Satisfaction improved over time. The extended follow-up for an average of 19 years gave exceptional insight into the long-term state for patients with this injury and provided reassurance that, although some long-term symptoms of intermittent pain and stiffness can be anticipated, these are usually reported to be self-limiting. The outcome for simple dislocation of the elbow was not universally benign, but patients did report overwhelmingly excellent functional outcomes and levels of satisfaction into the very long term.

Primary ligamentous repair is increasingly being considered and offered to patients with acute traumatic elbow instability. The excellent long-term results demonstrated in our study mean that primary surgery is likely not necessary for most patients with simple dislocation of the elbow.

Advanced studies with magnetic resonance imaging (MRI) have been used to help select patients with high-grade injuries for which it is anticipated that surgery may add value^12^, with the recognition that a simple elbow dislocation encompasses a spectrum of injury. In our practice, MRI is a useful adjunct but is not part of our routine assessment for all patients. The results of our study suggest that this pragmatic approach is appropriate for most patients.

There is some evidence that the osseous morphology of the ulnohumeral joint may contribute to the risk of recurrent elbow joint instability^13^. This may add to the argument that there could be a role for advanced imaging to identify patients with injuries in whom early surgery may be appropriate. Work to clearly define the patient groups or injury patterns that might benefit from surgical treatment would support appropriate and informed decision-making and ensure that the suggestion of surgery is appropriately targeted. It is possible that surgery may offer some advantages such as speedier recovery, earlier mobilization, or additional benefits for certain high-demand patients, but there is limited direct evidence to support this or to define exactly which patients or injuries should be considered for surgery.

We recognize that, in patients with injury patterns that are recognized as particularly high-energy and unstable, it may not be possible to achieve and maintain closed joint reduction; therefore, our protocol, as described above, would not be suitable. Anatomic and imaging studies have characterized a subgroup of posteromedial elbow dislocations in these patients^14^.

Despite the acknowledged potential for some selection or treatment bias and the retrospective nature of this study, the results provide valuable data on extended follow-up in patients with simple dislocation of the elbow. Our study provides helpful baseline data on the excellent very long-term outcomes following an established nonoperative treatment protocol and against which any potential benefits of surgery may be compared.
